# Inhibition of Nuclear Nox4 Activity by Plumbagin: Effect on Proliferative Capacity in Human Amniotic Stem Cells

**DOI:** 10.1155/2013/680816

**Published:** 2013-12-29

**Authors:** Marianna Guida, Tullia Maraldi, Elisa Resca, Francesca Beretti, Manuela Zavatti, Laura Bertoni, Giovanni B. La Sala, Anto De Pol

**Affiliations:** ^1^Department of Surgical, Medical, Dental and Morphological Sciences with Interest in Transplant, Oncology and Regenerative Medicine, University of Modena and Reggio Emilia, Via del Pozzo 71, 41100 Modena, Italy; ^2^Department of Obstetrics and Gynecology, Arcispedale Santa Maria Nuova, Viale Risorgimento 80, 42100 Reggio Emilia, Italy

## Abstract

Human amniotic fluid stem cells (AFSC) with multilineage differentiation potential are novel source for cell therapy. However, *in vitro* expansion leads to senescence affecting differentiation and proliferative capacities. Reactive oxygen species (ROS) have been involved in the regulation of stem cell pluripotency, proliferation, and differentiation. Redox-regulated signal transduction is coordinated by spatially controlled production of ROS within subcellular compartments. NAD(P)H oxidase family, in particular Nox4, has been known to produce ROS in the nucleus; however, the mechanisms and the meaning of this function remain largely unknown. In the present study, we show that Nox4 nuclear expression (nNox4) increases during culture passages up to cell cycle arrest and the serum starvation causes the same effect. With the decrease of Nox4 activity, obtained with plumbagin, a decline of nuclear ROS production and of DNA damage occurs. Moreover, plumbagin exposure reduces the binding between nNox4 and nucleoskeleton components, as Matrin 3. The same effect was observed also for the binding with phospho-ERK, although nuclear ERK and P-ERK are unchanged. Taken together, we suggest that nNox4 regulation may have important pathophysiologic effects in stem cell proliferation through modulation of nuclear signaling and DNA damage.

## 1. Introduction

Stem cells are characterized by a high capacity of self-renewal and differentiation. Through self-renewal, stem cells maintain the homeostasis of a stem cell pool; through differentiation, stem cells can give rise to terminal cells with diverse morphology and functions [1]. In tissues, most stem cells are in the quiescent state, and they are protected by special microenvironments (niches) [2]. The quiescence of stem cells may prevent the accumulation of DNA replication errors [3] and may facilitate resistance to many stressors [4]. The intracellular ROS level is a critical factor that regulates the quiescent status of mesenchymal stem cells (MSC) [5]. Similar to the low partial pressure of oxygen, low levels of ROS in niches are important for the stemness of MSC [6]. However, *in vitro* expansion of stem cells implies normoxic culture condition.

Indeed, MSC proliferative and colony formation capacity is significantly increased in normoxia. However, MSC expanded under normoxia show a threefold to fourfold increase in senescence, suggesting that hypoxia prevents oxidative stress-induced senescence and preserves MSC long-term self-renewal [7].

Accumulation of ROS is a common occurrence in senescent cells. Studies have shown that induction of ROS in senescent cells is involved in inhibiting proliferation [8]. On the other hand, intracellular accumulation of H_2_O_2_ in senescent human fetal MSCs termed placenta-derived multipotent cells (PDMCs) has been found, but the accumulation was not involved in inhibiting proliferation. Rather, H_2_O_2_ was involved in altering the differentiation potential of senescent PDMCs [9].

Various ROS-generating and ROS-degrading systems in different compartments of the cell seem to play an important role. The nucleus itself contains a number of proteins with oxidizable thiols that are essential for transcription, chromatin stability, and nuclear protein import and export, as well as DNA replication and repair [10]. Specific isoforms of glutathione peroxidases, glutathione S-transferases, and peroxiredoxins are enriched in nuclei, further supporting the interpretation that functions of the thiol-dependent systems in nuclei are at least quantitatively and probably also qualitatively distinct from similar processes in the cytoplasm [11].

ROS generation within the nucleus may have several important effects on cellular function. ROS can inactivate nuclear-localized phosphatases and thereby enhance kinase activation. For example, the oxidative inactivation of the nuclear phosphatase mitogen-activated kinase phosphatase 1 regulates ERK1/2 activation [12]. Excessive production of ROS could also lead to oxidative DNA damage.

In this point of view, the subcellular localization of NADPH oxidase isoform 4 (Nox4) is likely to be especially important, given its constitutive activity, unlike isoforms, such as Nox1 or Nox2, that requires agonist activation. However, its subcellular distribution remains controversial, at least in part attributable to the lack of sufficiently specific or characterized antibodies. Nox4 has been reported to be variably present in the ER [13, 14], mitochondria [15], cytoskeleton [16], plasma membrane [17], and nucleus [18] in different cell types.

Other unresolved questions include whether Nox4 utilizes NADPH or NADH as a substrate to produce O_2_^.^ [18, 19] and whether it primarily produces superoxide or hydrogen peroxide [18, 20].

More recently, endothelial nuclei have been shown to produce ROS that are, at least in part, Nox4 dependent [18, 21], but its subnuclear localization (within specific nuclear membranes) remains unclear [22]. Nuclear Nox4 has also been implicated in DNA damage resulting from both hemangioendothelioma formation [23] and hepatitis C infection [24].

NADPH oxidase Nox4 is a critical mediator in oncogenic H-RasV12-induced DNA damage response [25]. DNA damage response, detected by c-H2A.X foci analysis, leads to cell aging and subsequent senescence [26].

Anilkumar et al. [27] found that there is a nuclear-localized and functionally active splice variant of Nox4 (Nox4D) that may have important pathophysiologic effects through modulation of nuclear signaling and DNA damage. Interestingly, a significant proportion of nuclear Nox4D was localized to the nucleolus of vascular cells.

In this study, we investigated the role of Nox4-derived nuclear ROS on proliferative capacity of amniotic fluid stem cells (AFSC) since they can be considered representative of human stem cells, in view of their characteristics, such as the intermediate status between embryonic stem cells and adult stem cells.

Moreover, De Coppi and colleagues [28] described that AFSC can be directed into a wide range of cell types representing the three primary embryonic lineages of mesoderm, ectoderm, and definitive endoderm [29, 30]. Amniotic fluid is known to contain a heterogeneous population of cell types derived from fetal tissues and the amnion [31]. *In vitro* expansion of human stem cells is necessary to obtain a sufficient cell number to *in vivo* implant purpose, but it leads to senescence affecting proliferative and differentiation capacities. Thus, if the function of ROS is to enforce irreversible cellular senescence, the NADPH oxidase Nox4, as ROS-generating system, appears to be a potential nuclear ROS source.

Here, we show that a part of Nox4 localizes to the nucleus of AFSC, where the oxidase likely forms a functional complex with p22phox and produces ROS in the nucleoplasm. In addition, Nox4 seems to regulate DNA damage, constituting a part of oxidative stress. Here, we assessed the effect of plumbagin, a plant-derived naphthoquinone directly inhibiting Nox4 activity [32], on DNA damage and Nox4 nuclear interactions.

## 2. Materials and Methods

### 2.1. Cell Culture

Amniocentesis samples (5 back up flasks obtained from different donors) were provided by the Laboratorio di Genetica, Ospedale Santa Maria Nuova (Reggio Emilia, Italy). All samples were collected with informed consent of the patients (mother's age ≥ 35) according to Italian law and ethical committee guideline.

Human AFSC were isolated as previously described by De Coppi et al. [28]. Human amniocentesis cultures were harvested by trypsinization and subjected to c-Kit immunoselection by MACS technology (Miltenyi Biotec, Germany). AFSC were subcultured routinely at 1 : 3 dilution and not allowed to expand beyond the 70% of confluence. AFSC were grown in culture medium (*α*MEM supplemented with 20% fetal bovine serum (FBS), 2 mM L-glutamine, 100 U/mL penicillin, and 100 *μ*g/mL streptomycin) (all from EuroClone Spa, Italy) [33].

Cells were treated with plumbagin (5-hydroxy-2-methyl-1,4 naphthoquinone) or diphenyleneiodonium (Sigma Aldrich, St. Louis, MO,USA).

### 2.2. Cell Viability and Proliferation Assay

Viable cells were evaluated by the MTT assay, since the reduction of tetrazolium salts is widely accepted as a reliable way to examine cell viability/proliferation. Cells were incubated with 0.5 mg/mL MTT for 4 h at 37°C, as previously reported [34]. At the end of the incubation, purple formazan salt crystals were dissolved by adding the solubilization solution (isopropanol, 0.01 M HCl). The absorption at 570 nm was measured on a multiwell plate reader (Appliskan, Thermo-Fisher Scientific, Vantaa, Finland).

### 2.3. Cell Cycle Analysis

For detection and quantification of cell cycle distribution, samples containing 2–5 × 10^5^ cells were harvested by centrifugation, fixed in cold ethanol, and subjected to propidium iodide (Sigma Aldrich, St. Louis, MO, USA) flow cytometric assay. Total lysates, obtained as reported below, were subjected to Western blotting and revealed for anticyclin E (Santa Cruz Biotechnology, Santa Cruz, CA, USA).

### 2.4. Preparation of Cell Extracts

Cell extracts were obtained as described by Maraldi et al. [35]. Briefly, subconfluent cells were extracted by addition of AT lysis buffer (20 mM Tris-Cl, pH 7.0; 1% nonidet P-40; 150 mM NaCl; 10% glycerol; 10 mM EDTA; 20 mM NaF; 5 mM sodium pyrophosphate; and 1 mM Na_3_VO_4_) and freshly added Sigma Aldrich protease inhibitor cocktail at 4°C for 30 min. Lysates were sonicated, cleared by centrifugation, and immediately boiled in SDS sample buffer or used for immunoprecipitation experiments, as described below.

### 2.5. Immunoprecipitation and Electrophoresis

Immunoprecipitation was performed as reported by Cenni et al. [36]. Equal amounts of precleared lysates, whose protein concentration was determined by the Bradford method, were incubated overnight with anti-NOX4 (Novus Biologicals, CO, USA), antipan 14.3.3 (Santa Cruz Biotechnology, Santa Cruz, CA, USA) (3 *μ*g all). Then, the two samples were treated with 30 *μ*L of 50% (v/v) of protein A/G agarose slurry (GE Healthcare Bio-sciences, Uppsala, Sweden) at 4°C with gentle rocking for 1 h. Pellets were washed twice with 20 mM Tris-Cl, pH 7.0; 1% Nonidet P-40; 150 mM NaCl; 10% glycerol; 10 mM EDTA; 20 mM NaF; and 5 mM sodium pyrophosphate and once with 10 mM Tris-Cl, pH 7.4, boiled in SDS sample buffer, and centrifuged. Supernatants were loaded onto SDS-polyacrylamide gel, blotted on Immobilon-P membranes (Millipore, Waltham, MA, USA), and processed by Western blot with the indicated antibodies, detected by Supersignal substrate chemiluminescence detection kit (Pierce, Rockford, IL, USA). Quantitation of the signal was obtained by chemiluminescence detection on a Kodak Image Station 440CF and analysis with the Kodak 1D Image software.

### 2.6. Nuclei Purification

Human AFSC nuclei were purified as reported by Cenni et al. [37]. Briefly, to 5 × 10^6^ cells 400 *μ*L of nuclear isolation buffer was added (10 mM Tris-HCl, pH 7.8, 1% Nonidet P-40, 10 mM *β*-mercaptoethanol, 0.5 mM phenylmethylsulfonyl fluoride, 1 *μ*g/mL aprotinin and leupeptin, and 5 mM NaF) for 8 min on ice. MilliQ water (400 *μ*L) was then added to swell cells for 3 min. Cells were sheared by passages through a 22-gauge needle. Nuclei were recovered by centrifugation at 400 ×g at 4°C for 6 min and washed once in 400 *μ*L of washing buffer (10 mM Tris-HCl, pH 7.4, and 2 mM MgCl_2_, plus inhibitors as described earlier in the text). Supernatants (containing the cytosolic fractions) were further centrifuged for 30 min at 4000 ×g. Isolated nuclear and cytoplasmic extracts were finally lysed in AT lysis buffer, sonicated, and cleared by centrifugation.

### 2.7. Western Blot

The protocols of the western blot were performed as described by Hanson et al. [38].

Protein extracts, quantified by a Bradford Protein Assay (Bio-Rad Laboratories, CA, USA), underwent to SDS-polyacrylamide gel electrophoresis and were transferred to Immobilon-P membranes. The following antibodies were used: rabbit antipan 14.3.3, rabbit anticyclin E, rabbit anti-Nox4, rabbit anti-Ki67, Rabbit anti-ERK1/2, goat anti-Matrin 3, goat anti-actin, anti-p22phox (Santa Cruz Biotechnology, Santa Cruz, CA, USA) diluted 1 : 500, rabbit anti-p44/42 ERK1/2 (Cell Signalling Technology, Beverly, MA, USA), mouse anti-Tubulin (Sigma Aldrich St. Louis, MO, USA), rabbit anti-Nox4 (Abcam, Cambridge, UK), rabbit anti-Nox4 (Novus Biologicals, CO, USA), and mouse anti-pH2A (Ser139) (Millipore, Billerica, MA, USA) diluted 1 : 1000; peroxidase-labelled anti-rabbit, mouse, and goat secondary antibodies diluted 1 : 3000 (Pierce Antibodies, Thermo Scientific; Rockford, IL, USA). Ab dilution was performed in TBS-T pH 7.6 containing 3% BSA. The membranes were visualized using Supersignal substrate chemiluminescence detection kit (Pierce, Rockford, IL, USA). Antiactin antibody was used as control of protein loading.

### 2.8. Confocal Microscopy

Undifferentiated AFSC were fixed for 20 min in 4% ice-cold paraformaldehyde and then permeabilized with 0.1% Triton X-100 in ice-cold phosphate-buffered saline (PBS) for 5 min. Permeabilized samples were then blocked with 3% of bovine serum albumin (BSA) in PBS for 30 min at room temperature and incubated with primary antibodies (Abs). Rabbit anti-Nox4, goat anti-Matrin 3, rabbit anti-Ki67 (Santa Cruz, CA, USA) (diluted 1 : 50), and mouse anti-pH2A (Ser139) (Millipore, Billerica, MA, USA) (diluted 1 : 100), in PBS containing 3% BSA for 1 h at RT were used as primary antibodies (Ab). Secondary Ab was diluted 1 : 200 in PBS containing 3% BSA (goat anti-mouse Alexa 647, goat anti-rabbit Alexa 488, and donkey anti-goat Alexa 488). After washing in PBS, samples were stained with 1 *μ*g/mL DAPI in H_2_O for 1 min and then mounted with antifading medium (0.21 M DABCO and 90% glycerol in 0.02 M Tris, pH 8.0). Negative controls consisted of samples not incubated with the primary antibody, but only with the secondary antibody.

Confocal imaging was performed on a Nikon A1 confocal laser scanning microscope as previously described [39].

Spectral analysis was carried out to exclude overlapping between two signals or the influence of autofluorescence background on the fluorochrome signals, as previously shown [40]. The confocal serial sections were processed with ImageJ software to obtain three-dimensional projections, as previously described [41]. The image rendering was performed by Adobe Photoshop software.

### 2.9. Nuclear ROS Imaging

Nuclear ROS were detected with nuclear-localized fluorescent probe for H_2_O_2_, Nuclear Peroxy Emerald 1 (NucPE1) [42–45]. For all experiments, 5 *μ*M solutions of NucPE1 (from 5 mM stocks in DMSO) were made in PBS/glucose. The cells were then kept in an incubator (37°C, 5% CO_2_) during the course of all experiments. The probes were incubated for total 30 min. Fluorescence was measured on a multiwell plate reader (Appliskan, Thermo Scientific) using 488 nm filter for excitation and 535 nm filter for emission.

Confocal fluorescence imaging studies were performed with a Nikon A1 confocal laser scanning microscope. Excitation of NucPE1-loaded cells at 488 nm was carried out with an Ar laser and emission was collected at 535 nm. All images in an experiment were collected simultaneously using identical microscope settings. Image analysis was performed in Image J.

### 2.10. Statistical Analysis

*In vitro* experiments were performed in triplicate. For quantitative comparisons, values were reported as mean ± SD based on triplicate analysis for each sample. To test the significance of observed differences between the study groups, unpaired Student's *t*-test was applied. A *P* value < 0.05 was considered to be statistically significant.

## 3. Results and Discussion

### 3.1. Nox4 Expression in AFSC

**Figure 1:**
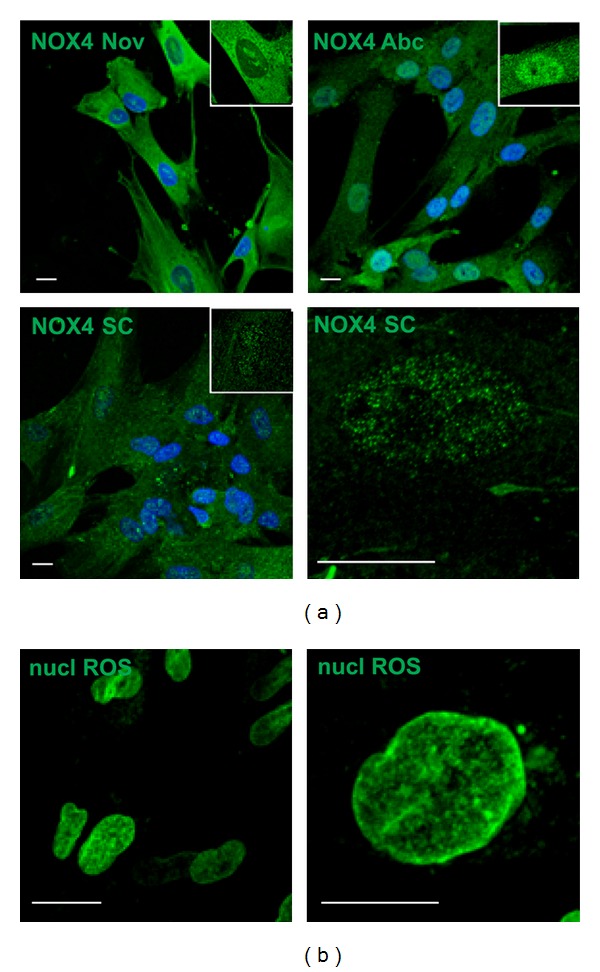
Immunofluorescence analysis of Nox4 expression and nuclear ROS in AFSC.  (a) Representative images showing superimposing between DAPI (blue) and Nox4 (green) signals obtained with antibody from Novus (Nov), Abcam (Abc), and Santa Cruz (SC). Inside all the three images, a square showing magnification of a cell with only the Nox4 signal in green is present. The highest magnification of one nucleus is shown for Santa Cruz labelling. (b) Representative images showing staining with nuclear ROS probe (Nuclear peroxy Emerald 1). Scale bar: 10 *μ*m.

At first we noticed that amniotic fluid stem cells express NADPH isoform 4 Nox4 both into the nucleus and in the cytoplasm, unlike Nox1 and Nox2 (data not shown). We tested different antibodies directed to Nox4 protein in order to verify this observation. In fact, in the literature, a homemade antibody anti-Nox4 is often used (polyclonal Nox4 antibodies by the Lambeth and Shah groups are the most frequently used) (see review [46]). On the other hand, all the employed antibodies revealed a variable percentage of cells with a nuclear localization of Nox4 (Figure 1(a)) and this data is consistent with other studies in which Nox4 has been found in nuclear or perinuclear area [18, 25, 47, 48]. By using antibodies from Santa Cruz, Abcam, or Novus, we can see a signal mostly localized inside the nuclei, not in the nuclear envelope, as demonstrated in mouse liver cells [48]. Regarding the staining obtained with Abcam antibody, all the nucleoplasm is marked except for nucleoli. Novus antibody binds other perinucleolar domains, while Santa Cruz antibody shows a more punctate pattern.

In order to investigate the NADPH oxidase activity inside nuclei, we used a nuclear selective probe for H_2_O_2_, nuclear Peroxy Emerald 1 (Figure 1(b)). Detection of nuclear ROS production with this probe demonstrates that ROS sources inside the nuclei have a punctate pattern similar to the one of Nox4 from Santa Cruz. Therefore, for immunofluorescence analysis, we utilized this antibody.

**Figure 2:**
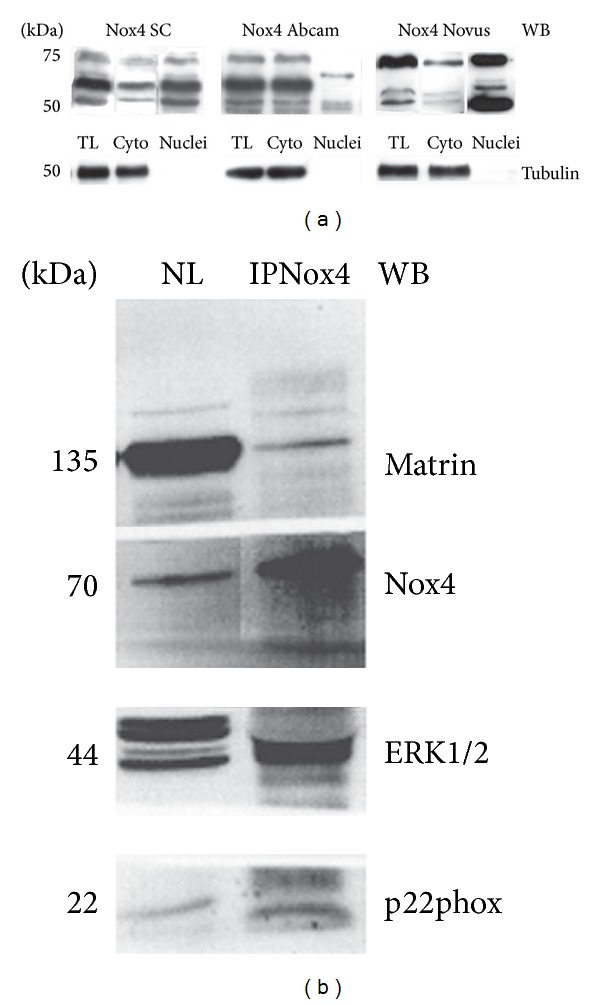
Nox4 expression in subcellular compartment of AFSC. (a) Representative images of Western blot analysis of total lysates (TL), cytosol (cyto) and nuclear fractions (nuclei) of AFSC revealed with different Nox4 antibodies: Santa Cruz (SC), Abcam (Abc), and Novus (Nov). Tubulin is shown as marker of nuclei purification. (b) Western blot analysis of nuclear lysate (NL) and immunoprecipitation experiment of NL with Nox4 antibody from Novus revealed with anti-Matrin, anti-Nox4, anti-ERK1/2, and anti-p22phox. Presented data are representative of three independent experiments.

The same analysis has been performed for Western blot experiments (Figure 2(a)). Total lysates, cytosol, and nuclear fractions and the three antibodies reported above were tested.

WB analysis with all the antibodies of nuclear and cytoplasmic fractions confirms Nox4 presence both in cytosol and nuclei. Incubation with all the three antibodies reveals a triple band between 50 and 75 kDa, even if the band intensity is different depending on the antibody. In WB, the antibody from Novus seems to recognize the nuclear Nox4 (nNox4) better than others, since the bands in nuclear protein fraction are the most intense (Figure 2(a)).

The Novus antibody works also in immunoprecipitation experiment.  Figure 2(b) shows nuclear extracts (NL) used for coimmunoprecipitation analysis with anti-Nox4 (IPNox4). This experiment confirms the presence of Nox4 in nuclear proteins showing the interaction between Nox4 and the modulatory subunit p22phox. Furthermore, Nox4 seems to be linked with nuclear matrix protein, Matrin3, and with the mitogen-activated protein kinase ERK1/2, suggesting a direct role in nuclear MAPK signaling regulation. It has been previously demonstrated that ERK activation (phosphorylation) occurs downstream from the Nox4 pathway: in particular through the Ras activation in endoplasmic reticulum of Human umbilical vein endothelial cells [49] or by a Src/caveolin-mediated activation in renal tubular cells [50].

### 3.2. Modulation of Nox4 Presence into the Nuclei

**Figure 3:**
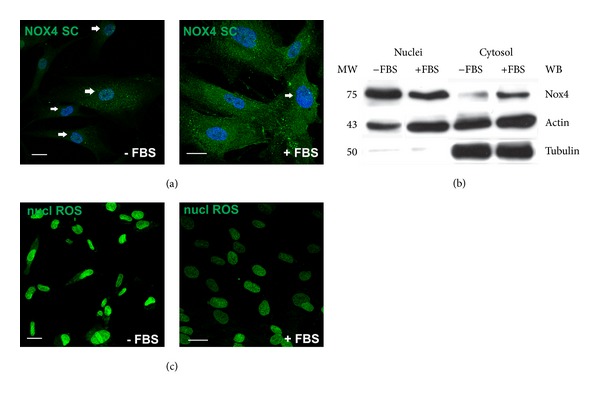
Effect of serum presence on Nox4 localisation end nuclear ROS production. (a)  Representative images showing superimposing between DAPI (blue) and Nox4 SC (green) signals of AFSC cultures in the presence of absence of serum (FBS). Arrows indicate Nox4 staining in the nuclei. (b) Representative images of Western blot analysis revealed with Nox4 from Novus of cytosol and nuclei fractions of AFSC grown in the presence or absence (for 24 hours) of FBS. Actin and tubulin detection were performed in order to show the amount of protein loaded in each line and the nuclear fraction purity, respectively. Presented data are representative of three independent experiments. (c) Representative images showing staining with nuclear ROS probe (Nuclear peroxy Emerald 1) of AFSC with or without serum. Scale bar: 10 *μ*m.

The ERK cascade is involved in cellular proliferation, differentiation, and survival. In fact nuclear translocation of ERK1 and ERK2 is critical for both gene expression and DNA replication induced by growth factors [51]. Moreover, in the nucleus, ERK phosphorylates an array of targets, including transcription factors involved in multiple aspects of growth control. Based on these considerations, we investigated the effect of proliferation stimuli on Nox4 localization in AFSC. AFSC are usually grown in the presence of 20% serum in order to induce the best proliferation rate. Serum starvation (−FBS) in fact affects Nox4 distribution within the cell. As demonstrated by WB and IF analysis shown in Figures 3(a) and 3(b), the nuclear portion of Nox4 (nNox4) increases in AFSC cultured without FBS for 24 hours. In fact, confocal images show the Nox4 punctate pattern in the majority of nuclei in–FBS sample. Serum deprivation induces also a decrease in Nox4 staining in the cytosol (Figures 3(a) and 3(b)), compared to +FBS sample.

This observation is consistent with data obtained with the fluorogenic probe assay for nuclear ROS. In fact the number of nuclei strongly stained with the probe is higher in culture −FBS (Figure 3(c)). The nuclear ROS quantification shows that the increase of fluorescence intensity in the absence of FBS is around 50%.

**Figure 4:**
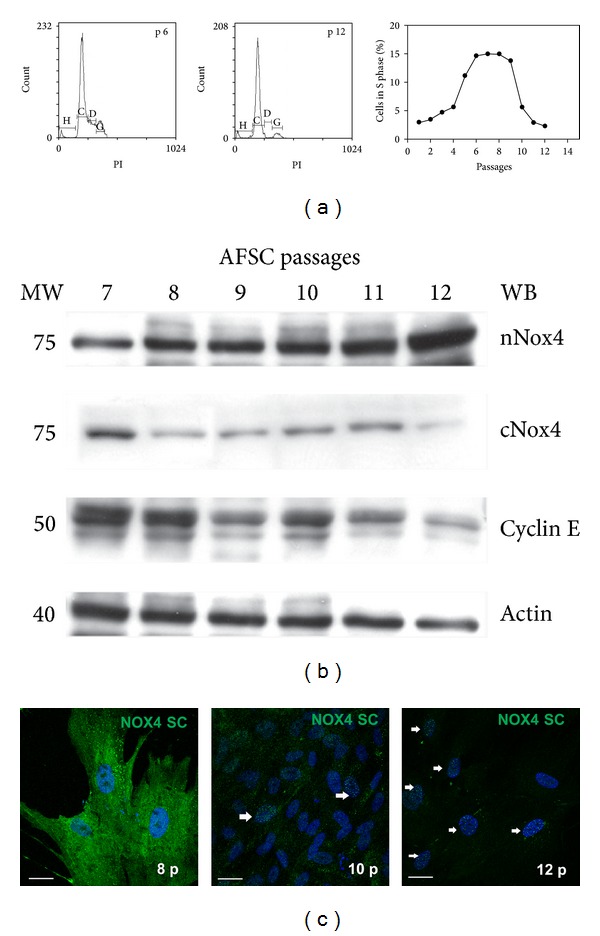
Nox4 localisation during culture passages. (a) Cytofluorimetric analysis with propidium iodide (PI) of AFSC in culture from passage 1 to 12. Representative FACS analysis of cells at passages 6 and 12. The graph shows the percentage of cells on S phase. (b) Representative images of Western blot analysis of cytosol and nuclei of AFSC from passages 7 to 12 and revealed with Nox4 from Novus, Cyclin E, and actin. Presented data are representative of three independent experiments. (c) Representative images showing superimposing between DAPI (blue) and Nox4 SC (green) signals of AFSC cultures at passages 8, 10, and 12. Arrows indicate Nox4 staining in the nuclei. Scale bar: 10 *μ*m.

Amniotic fluid stem cells, after a week of slow proliferation in culture, can be passaged at 70% confluence every two to three days. As mesechymal stem cells, they can rapidly react to changes of the culture environment during expansion. In particular, MSC have been proved to gradually become senescent and decrease their proliferative capacity while in culture. The proliferation curve of AFSC in our experimental condition is shown in Figure 4(a), where the percentage of cells in S phase is graphed. After the first 3-4 passages of slow growth, an increase in proliferation occurs up to a plateau state around the 8th passage; hence, the number of cells able to divide decreases quickly.

Western blot analysis of AFSC during late passages (Figure 4(b)) shows that nuclear Nox4 presence dramatically increases at cell cycle arrest, decreasing the cytosol. Indeed confocal images show the Nox4 punctate pattern in the majority of nuclei in sample at 12th passage. In fact, while Cyclin E expression decreases at 12th passage, indicating a stop in proliferation, Nox4 raises in nuclear fraction. The same trend can be observed by IF analysis, as shown in Figure 4(c).

### 3.3. Effect of Nox4 Inhibition on AFSC Proliferation

**Figure 5:**
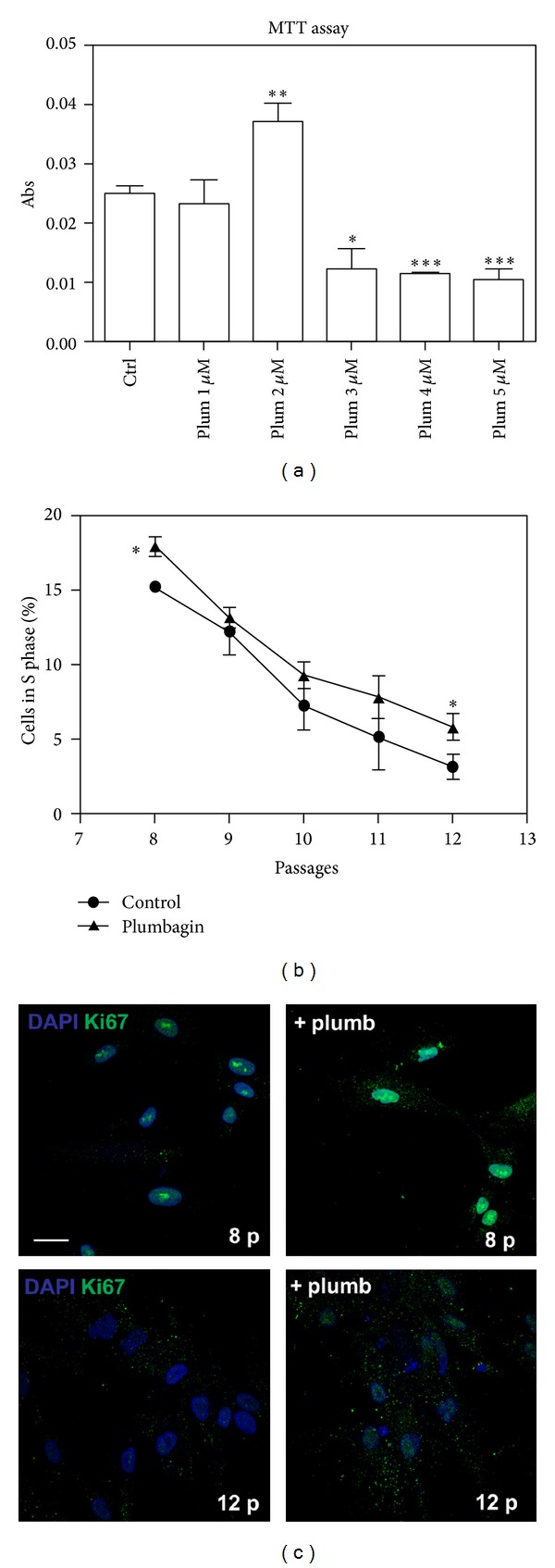
Effect of plumbagin on AFSC proliferation. (a) AFSC (between the 6th and at 8th passages) were incubated for 24 hours with increasing concentration of plumbagin (0–5 *μ*M). Cell viability was determined with the MTT test, as described in the Material and Methods section. ***P < 0.0001; **P < 0.01; *P < 0.05 significantly different from control cells. (b) Cytofluorimetric analysis with propidium iodide (PI) of AFSC in culture from passages 8 to 12 in the presence or absence of 2 *μ*M plumbagin. The graph shows the percentage of cells on S phase. Presented data are representative of three independent experiments. *P < 0.05 significantly different from control cells. (c) Representative images showing superimposing between DAPI (blue) and Ki67 (green) signals of AFSC cultures at passages 8 and 12 in the presence or absence of 2 *μ*M plumbagin for 24 hours. Scale bar: 10 *μ*m.

In order to evaluate the effects of Nox4 inhibition on cell viability/proliferation, we used plumbagin, a plant-derived naphthoquinone, directly interacting with Nox4 and inhibiting its activity [31]. MTT test shows that 1-2 *μ*M plumbagin does not affect negatively cell viability, but rather, at 2 *μ*M concentration plumbagin is able to significantly improve cell proliferation. At higher concentrations, plumbagin decreases AFSC viability around 50–60% after 1 day of incubation (Figure 5(a)); therefore, these concentrations were not chosen for the reported experiments.

Moreover, late passages (8–12 p) AFSC cultured in the presence of 2 *μ*M plumbagin display a better proliferation trend, as shown in graph of phase S cells (Figure 5(b)), even if not always in a significant manner. The same indication is provided by IF experiment of Ki-67 staining during culture passages (Figure 5(c)). The expression of the human Ki-67 protein is strictly associated with cell proliferation. The number of nuclei positive for Ki-67 is dramatically reduced in sample of late passage. In the presence of plumbagin, the staining of Ki-67 is higher both at 8th and 12th passages, compared to control samples.

**Figure 6:**
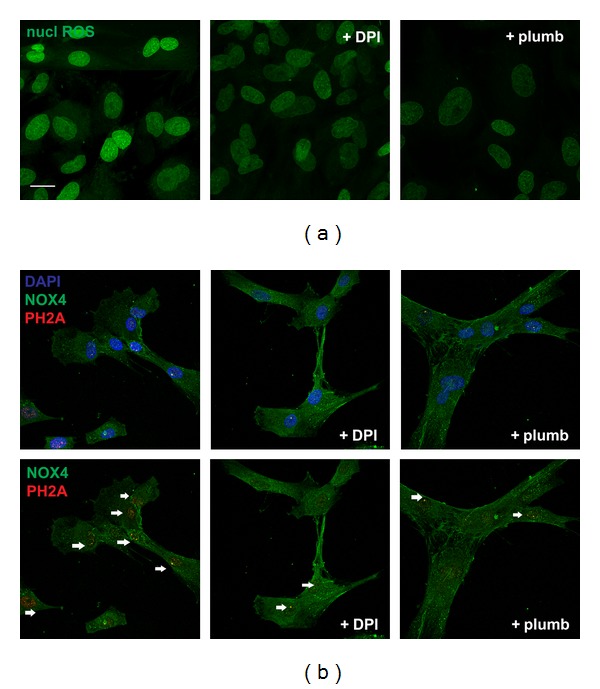
Effect of Nox4 inhibition on nuclear ROS production and DNA damage. (a) Representative images showing staining with nuclear ROS probe (Nuclear peroxy Emerald 1) of AFSC in the presence or absence of 2 *μ*M plumbagin or 1 *μ*M DPI for 24 hours. Scale bar: 10 *μ*m. (b) Representative images showing superimposing between Nox4 SC (green) and PH2A (red) signals with or without DAPI (blue) of AFSC in the presence or absence of 2 *μ*M plumbagin or 1 *μ*M DPI for 24 hours. Arrows indicate the PH2A staining in the nuclei. Scale bar: 10 *μ*m.

Even if the use of Nox4 synthetic or natural inhibitors, diphenyleneiodonium (DPI) and Plumbagin, is not directed to the nuclear part of Nox4, as demonstrated by the fluorogenic probe assay, the Nox4 activity inhibition reduces the nuclear ROS production (Figure 6(a)). The nuclear ROS quantification shows that the decrease of fluorescence intensity in the presence of DPI or plumbagin is around 50% for both. This effect can regulate cell proliferation also through a modulation of DNA stability. In fact, ROS can also activate both cell survival and senescence pathways depending on its concentration and localization [52–54]. To assess whether nNox4-generated ROS can induce nuclear DNA damage, we studied nuclear H2A foci. It has been shown recently that the status of H2AX phosphorylation is crucial to determine whether cells will survive after DNA damage [55].

The ROS production decrease, obtained with synthetic or natural Nox4 inhibitors (DPI and Plumbagin), reduces the staining for the phosphorylated form of histone H2AX, marker of oxidative stress derived-DNA damage (Figure 6(b)).

### 3.4. Effect of Plumbagin on Nox4 Nuclear Interaction

**Figure 7:**
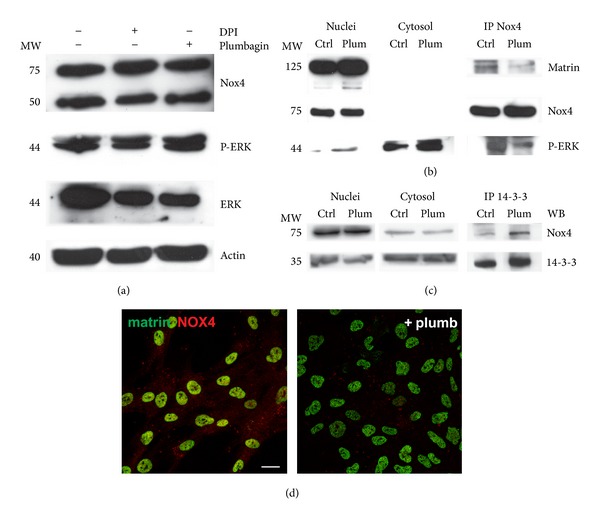
Effect of plumbagin on Nox4 nuclear interaction. (a)  Representative images of Western blot analysis of total lysates of AFSC treated with 2 *μ*M plumbagin or 1 *μ*M DPI for 24 hours. Membranes were revealed with anti-Nox4, anti-PERK1/2, and antiactin. (b) Subcellular fractions obtained with the plumbagin treatment were prepared for immunoprecipitation analysis. Nuclear lysate (NL) were immunoprecipitated with Nox4 antibody from Novus then revealed with anti-Matrin, anti-Nox4, and anti-PERK1/2. (c) Nuclear lysate (NL) were immunoprecipitated with anti-14-3-3 then revealed with anti-Nox4 and anti-14-3-3. All presented data are representative of three independent experiments. (d) Representative images showing superimposing between Matrin 3 (green) and Nox4 (red) signals of AFSC in the presence or absence of 2 *μ*M plumbagin for 72 hours. Scale bar: 10 *μ*m.

We investigated the effect of Nox4 inhibition on the previously observed nuclear Nox4 binding network (Figure 2(b)). The Nox4-derived ROS decrease, obtained with DPI or plumbagin incubated for 24 hours, reduces the expression of ERK in total lysates, but the P-ERK level remains unchanged (Figure 7(a)). Coimmunoprecipitation experiment for Nox4 shows that, in the presence of plumbagin, Nox4 binding with P-ERK decreases (Figure 7(b)). This data confirms the link between Nox4 and ERK1/2 within the nucleus, as shown also in VSMC where Nox4 specifically increased nuclear phosphorylated ERK1/2 [27] but emphasizes the direct association and the modulation of this nuclear kinase in the active form by the ROS source, Nox4.

Also the interaction with some nucleoskeleton molecules seems to be modulable by Nox4 inhibition. In fact, CoIP for Nox4 shows a decrease in matrin 3 binding in sample treated with plumbagin (Figure 7(b)). Matrin 3, an abundant protein of the internal nuclear matrix, has been linked to a variety of functional events. While many sites occurred proximal to nucleoli, no significant staining was detected within the nucleolar interior. On the other hand, matrin 3 has been demonstrated to bind DNA at sites termed scaffold/matrix attachment regions to regulate gene expression through interactions with chromatin remodeling. Matrin 3 has been involved also in transcriptome machinery through RNA processing and structural organization [56]. Matrin 3 could be a docking site where nuclear ROS signaling may exert its function on transcription/pre-mRNA modulation in specific nuclear domains.

14-3-3 proteins have the ability to bind a multitude of functionally diverse signaling proteins, including kinases, phosphatases, playing important roles in a wide range of vital regulatory processes, such as mitogenic signal transduction and cell cycle control.

There are common themes by which 14-3-3 proteins regulate different signaling pathways. 14-3-3 can control the location of proteins by preventing nuclear import/export or membrane translocation or both. Moreover, 14-3-3 may compete with other signaling proteins for binding on the target protein and may modulate its substrate from ubiquitination and degradation [57].

The effect of Nox4 activity inhibition by plumbagin could cause a Nox4 sequestration by 14-3-3 binding, as shown by CoIP experiment for 14-3-3 (Figure 7(c)). In fact, a longer incubation (72 h) with plumbagin induces a decrease in Nox4 presence inside nuclei, while matrin 3 pattern is unchanged (Figure 7(d)). Indeed, the image shows the double staining for matrin3 (green) and Nox4 (red). In the control case, the superimposing produces an orange staining, indicating the presence of high red signal, while in the sample with plumbagin the superimposing causes only a green staining, due to the anti-Matrin 3 labeling.

## 4. Conclusions

We observed that Nox4 isoform is expressed in human AFSC and, interestingly, it localizes into the nucleus. Confocal analysis demonstrates Nox4 presence in nucleoplasm domains, not only in nuclear membranes, suggesting that Nox4 could be involved in regulating DNA-mRNA processing machinery by ROS production in specific nuclear area. During culture passages up to cell cycle arrest, AFSC exhibit a proliferation rate inversely coupled with Nox4 presence into the nuclei. Furthermore, the serum starvation causes the same effect. Moreover, immunoprecipitation analysis demonstrated that Nox4 interacts with ERK signaling, suggesting a role in nuclear signaling pathways.

Inhibition of Nox4 activity, obtained with plumbagin, induces a decline of nuclear ROS production and of DNA damage. Moreover, plumbagin exposure reduces the binding between nNox4 nucleoskeleton components. The same effect was observed also for the binding with phospho-ERK, although nuclear ERK and P-ERK are unchanged. A longer incubation with plumbagin may modulate Nox4 nuclear expression, by controlling the protein localization or/and a degradation pathway involvement. Taken together, we suggest that nNox4 regulation may have important pathophysiologic effects in stem cell proliferation through modulation of nuclear signaling and DNA damage.

## Abbreviations

AFSC: Amniotic fluid stem cells
BSA: Bovine serum albumin
DABCO: 1,4-Diazabicyclo(2.2.2)octane
DAPI: 4′,6-Diamidino-2-phenylindole
DPI: Diphenyleneiodonium
EDTA: Ethylenediaminetetraacetic acid
MSCs: Mesenchymal stem cells
PBS: Phosphate buffered saline
TBS: Tris-buffered saline
TxTBS: Triton-X-100.
